# Editorial: Nutrigenetics and nutrigenomics in livestock

**DOI:** 10.3389/fgene.2026.1960311

**Published:** 2026-08-25

**Authors:** Guilherme Henrique Gebim Polizel, Wellison J. S. Diniz, Luiz F. Brito, Miguel Henrique de Almeida Santana

**Affiliations:** 1 Department of Animal Science, School of Animal Science and Food Engineering, University of São Paulo, Pirassununga, Brazil; 2 Department of Animal Sciences, College of Agriculture, Auburn University, Auburn, AL, United States; 3 Department of Animal Sciences, Purdue University, West Lafayette, IN, United States

**Keywords:** gene expression, genomics, microbiome, multi-omics, nutritional genomics, proteomics, transcriptomics

The integration of nutrition, high-throughput phenotyping, and molecular sciences has become essential for understanding phenotypic and metabolic processes in livestock and for advancing the field of animal science. Concepts such as nutrigenetics and nutrigenomics have emerged to describe the interactions between nutrients and genomic variability and their influence on gene expression, metabolism, and animal performance (Van Ommen, 2004; Mutch et al., 2005). This has revolutionized nutritional science and research, as it has allowed scientists to gain deeper insights into how different nutrients affect the molecular mechanisms underlying growth, nutrient efficiency, health, reproduction, and production. Consequently, the combination of nutrition and molecular biology data is becoming one of the most important tools for designing precision feeding programs (Giannico et al., 2026).

In the current Research Topic, we collected eight original research articles and one literature review, highlighting the progress of nutritional genomics and its applications to livestock science. The main objective of this Research Topic collection is to provide an overview of the most recent applications of omics technologies in animal nutrition studies and to enhance our understanding of gene and metabolic regulation, the microbiome, multi-omics, genome architecture, and their crosstalk with phenotypic variation underlying animal production, reproduction, and environmental adaptation in livestock.

The article by Wang et al. investigated the effects of dietary tyrosine supplementation on feather pigmentation and the feather follicle transcriptome in H-line chickens. The authors demonstrated that a diet containing 1.0% tyrosine, provided during 40 days, significantly increased melanin deposition in the feathers. Additionally, they identified differentially expressed genes involved in melanogenesis and reported over-represented signaling pathways, including MAPK and Wnt. The study highlighted *EDNRB2* as a potential key gene regulator of tyrosine-induced pigmentation, providing important insights into the molecular mechanisms controlling feather color in chickens.

In beef cattle, Furlan et al. investigated the long-term effects of maternal nutrition during gestation on the offspring’s ruminal fluid microbiome, revealing changes in specific bacterial taxa and metabolic pathways associated with nutrient utilization. Complementarily, Polizel et al. evaluated the effects of prenatal nutrition on skeletal muscle mRNA isoforms and alternative splicing in beef cattle offspring. Using RNA sequencing, the authors demonstrated that maternal protein-energy supplementation, particularly when provided throughout gestation, induced substantial changes in transcript expression and usage. Isoform switching was reported in genes related to amino acid transport, cytoskeletal organization, and muscle regeneration. These findings highlight persistent molecular programming effects that extend beyond gene-level regulation. In addition, Brown et al. used an integrative transcriptomic and proteomic approach to investigate molecular differences between fertile and subfertile beef heifers using peripheral white blood cells collected before artificial insemination. The authors identified genes and proteins associated with cell cycle regulation, metabolism, immune response, and hormone signaling pathways. Network analyses also revealed distinct regulatory and potential epigenetic patterns between fertility-based groups, highlighting peripheral blood as a promising non-invasive source of biomarkers for assessing reproductive performance in beef cattle.

Costa et al. provided a comprehensive overview of rumen-protected methionine supplementation in dairy and beef cattle. The literature review discussed methionine metabolism, its role as a methyl donor, and current strategies to protect methionine from ruminal degradation. In addition, the authors summarized evidence regarding the effects of rumen-protected methionine on milk production and composition, growth performance, reproduction, immune function, fetal development, and overall animal health. Collectively, the review provided an updated perspective on the application of this nutritional technology in ruminant production systems. Also, Esfahani et al. investigated the functional and genetic dynamics of the bovine *DGAT1* gene using an integrated approach that combined structural bioinformatics, molecular docking, genotyping, and gene expression analyses. The authors identified significant associations between *DGAT1* polymorphisms and milk fat content, with the KK genotype showing higher *DGAT1* expression and greater milk fat levels in Holstein cows. Additionally, the study identified lidocaine as a potential *DGAT1* modulator, offering new perspectives on lipid metabolism and on developing genetic and biochemical strategies to improve milk quality in dairy cattle.

In sheep, Zhang et al. evaluated calcium propionate supplementation as a silage additive for corn stover silage quality. The authors demonstrated improvements in silage fermentation quality, beneficial bacterial populations, nutrient digestibility, and growth performance of Hu lambs, highlighting a practical strategy for enhancing the utilization of low-quality forage resources. In another ovine study, González-Montero et al. reported the first development of ovine hepatic organoids. They demonstrated that this *in vitro* platform preserves key structural and metabolic characteristics of liver tissue and responds to nutrient supplementation, establishing a promising model for investigating nutrient-gene interactions and reducing the use of experimental animals in ruminant nutrition research.

Lastly, Gervásio et al. investigated the genomic architecture underlying feeding-behavior traits in pigs using genome-wide association studies and integrative bioinformatics analyses. The authors identified genomic regions, candidate genes, and regulatory factors, including *STAT3*, *STAT5A*, *STAT5B*, *RARA*, and *SMAD4*, associated with feeding behavior and metabolic regulation. These findings contribute to a better understanding of the genetic mechanisms that influence feed efficiency in pigs and provide valuable molecular insights to improve breeding strategies, production efficiency, and sustainability in pig production systems.

Overall, this Research Topic highlights the growing integration of nutrition and molecular sciences as a powerful approach to understanding the biological mechanisms regulating animal performance, health, and productive efficiency. The studies presented herein demonstrate how multi-omics technologies and emerging *in vitro* models are advancing our understanding of nutrient-gene interactions and the molecular pathways that underlie important phenotypic traits in livestock. Collectively, these contributions provide valuable insights into precision nutrition and sustainable livestock production, encouraging further interdisciplinary research to address future challenges in animal science.

## References

[B1] Giannico F. Carbonara C. Caputi Jambrenghi A. Ragni M. Salem A. Z. M. Tarricone S. (2026). Sensor-based precision feeding systems in animal production: technologies and applications. Animals 16 (9), 1333. 10.3390/ANI16091333 42121752 PMC13162990

[B2] Mutch D. M. Wahli W. Williamson G. (2005). Nutrigenomics and nutrigenetics: the emerging faces of nutrition. FASEB J. 19, 1602–1616. 10.1096/fj.05-3911rev 16195369

[B3] Van Ommen B. (2004). Nutrigenomics: exploiting systems biology in the nutrition and health arenas. Nutrition, 20 (1) ,4–8. 10.1016/j.nut.2003.09.003 14698007

